# Suppression of oral cancer by induction of cell cycle arrest and apoptosis using *Juniperus communis* extract

**DOI:** 10.1042/BSR20202083

**Published:** 2020-09-07

**Authors:** Ching-Chang Lee, Chih-Yen Hsiao, Shan-Chih Lee, Xiao-Fan Huang, Kai-Fu Chang, Ming-Shih Lee, Ming-Chang Hsieh, Nu-Man Tsai

**Affiliations:** 1 Division of Gastroenterology, Department of Internal Medicine, Kaohsiung Armed Forces General Hospital, Kaohsiung 80284, Taiwan, R.O.C.; 2 Department of Medical Laboratory Science and Biotechnology, School of Medicine and Health Sciences, FooYin University, Kaohsiung 83102, Taiwan, R.O.C.; 3 Division of Nephrology, Department of Internal Medicine, Chia-Yi Christian Hospital, Chia-Yi 60002, Taiwan, R.O.C.; 4 Department of Hospital and Health Care Administration, Chia Nan University of Pharmacy and Science, Tainan 71710, Taiwan, R.O.C.; 5 Department of Medical Imaging and Radiological Sciences, Chung Shan Medical University, Taichung 40201, Taiwan, R.O.C.; 6 Department of Medical Imaging, Chung Shan Medical University Hospital, Taichung 40201, Taiwan, R.O.C.; 7 Institute of Medicine, Chung Shan Medical University, Taichung 40201, Taiwan, R.O.C.; 8 Department of Medical Laboratory and Biotechnology, Chung Shan Medical University, Taichung 40201, Taiwan, R.O.C.; 9 Clinical Laboratory, Chung Shan Medical University Hospital, Taichung 40201, Taiwan, R.O.C.

**Keywords:** 5-Fluorouracil (5-FU), cell apoptosis, cell cycle, Juniperus communis, Oral cancer, synergistic effect

## Abstract

The oral cancer incidence rate is slowly increasing and is now the fifth leading cause of cancer-related death due to its high metastasis and recurrence rate. *Juniperus communis* is used as a traditional Chinese medicine and has been proven to have anti-cancer activity against neuroblastomas. In the present study, we further investigated the anti-cancer mechanisms of *J. communis* extract (JCo) on oral cancer and evaluated the synergistic effects of JCo combined with 5-fluorouracil (5-FU). We found that JCo inhibited oral cancer cell growth, and that JCo might be less cytotoxic to normal cells than to cancer cells. After JCo treatment, cell cycle arrest was observed at the G_0_/G_1_ phase through modulation of p53/p21 and Rb signaling. JCo also caused an increase in the sub-G_1_ phase and cell apoptosis via the intrinsic and extrinsic apoptosis pathways. JCo combined with 5-FU presented a synergistic effect to reduce cell viability. In conclusion, JCo inhibited oral cancer cell growth by inducing cell cycle arrest and activating cell apoptosis, and JCo significantly synergized with 5-FU. JCo might have the potential to be an adjuvant and a new therapeutic drug for oral cancer treatment.

## Introduction

Oral cancer is part of a group commonly referred to as head and neck cancers. According to cancer statistics, oral cancer is common in Taiwan with a high incidence rate in males [1–3]. The 5-year survival rate of patients with advanced oral cancer is 32.8%, and recurrence rate in the 2 years after chemotherapy treatment results in a high death rate, in United States [4–7]. In the early stage of oral cancer, the main form of treatment is surgery, and patients with advanced cancer or who are unable to undergo surgery are usually treated with combination therapy. Although the response rate is approximately 50–80% after chemotherapy due to drug resistance, patients may suffer from serious side effects such as hand-foot syndrome or organ damage [8,9]. Hence, despite the advances in cancer therapy, better solutions are needed to treat oral cancer.

With the increasing incidence of cancer, natural products have received more attention for use in cancer therapy and prevention medicine and have been used to cure or alleviate many kinds of diseases [10,11]. Many chemotherapy drugs originate from natural plants, such as Taxol, which is a compound extracted from *Taxus brevifolia*, and is used to treat metastatic ovarian or breast cancer [12]. Moreover, these natural products have shown potential in stopping or reversing tumorigenesis via regulation of the cell cycle, induction of apoptosis, or even targeting key molecular pathways to prevent tumor growth [13]. The cell cycle is regulated mainly by cyclin and cyclin-dependent kinase; however, cell cycle is a common loss-regulated in cancerous cells, leading to uncontrolled cell proliferation [14,15]. Moreover, apoptosis is a process of serial caspase cascade activation results in cell death without inducing a strong immune response that is preferred to develop as chemodrugs for clinical treatment [16–19]. As a result, natural products are regarded as a potential source of cancer drug therapy without the toxic side effects.

*Juniperus communis* is a plant that is used as a traditional medicine for its antiseptic, contraceptive and diuretic properties, and as an appetizer or flavoring agent. Recently, scientists have found that *J. communis* has anti-inflammatory, anti-diabetic, antioxidant and anti-microbial activities [20]. Moreover, *J. communis* has been reported to inhibit breast cancer proliferation [21] and neuroblastoma [22]; however, the inhibitory potential of *J. communis* extract (JCo) on oral cancer was still not clear. Furthermore, 5-Fluorouracil (5-FU) is a chemotherapeutic drug which is a pyrimidine analog, and its metabolite can incorporate into RNA and DNA, or inhibit thymidylate synthetase [23]. 5-FU has been widely used alone or in combination with other anticancer agents and/or radiotherapy for treating various types of cancer, such as head and neck, and colorectal cancer. 5-FU [24,25]. In the advanced oral cancer, 5-FU is usually administered in combination with cisplatin and the combined treatment would increase adverse effects due to its toxic effects. Hence, the aim of the present study was to illuminate the anticancer acidity of JCo and described the combinational potential of JCo plus 5-FU on oral cancer cell.

## Materials and methods

### Cell culture and reagents

The human gingival squamous cancer cell line, OECM-1, was developed by Dr C.Y. Yang and Dr C.L. Meng [26], culturing in DMEM/F12 supplement with 0.1% hydrocortisone and had performed the FemtoPath TP53 exon 8 Primer Set (HongJing Biotech., New Taipei City, Taiwan). The normal cell lines, SVEC (Mouse vascular endothelial cell) and MDCK (*Canis* kidney epithelial cell), were purchased from the American Type Culture Collection (Rockville, MD, U.S.A.) and cultured in DMEM. All media were supplemented with 10% fetal bovine serum, 1% sodium pyruvate, 1% HEPES buffer solution, and 1% penicillin-streptomycin. All cell culture reagent was purchased from Gibco (Grand Island, N.Y., U.S.A.). Cells were incubated in a 95% humidity and 5% CO_2_ atmosphere at 37°C.

### Preparation of *Juniperus communis* extract (JCo extract)

*Juniperus communis* fruit was freshly obtained from Nepal and performed steam distillation to gain the product that divided two layers, one was aqueous layer and the other was lipid layer, which was *J. communis* extract (JCo extract) and utilized throughout the study. The detail extraction flowchart was first tested in our lab with small-scale. A 2-L steam distillation steel apparatus unit was containing 400 ***g*** of *J. communis* fruits and the generated steam (flow rate: 7.2 ml/min) with 100–105°C was passed through plant material for 100 min. After that, the large scale of JCo extract was commissioned by Phoenix (New Jersey, U.S.A.). JCo was dissolved in DMSO (2%) and diluted with fresh medium before each experiment.

### MTT assay

Cell viability was assessed using the MTT assay. OECM-1 cells were cultured overnight at an appropriate density (5 × 10^3^ cells/100 μl) in a 96-well culture plate and treated for 24, 48 and 72 h in the presence of JCo (0–200 μg/ml). After the JCo was removed, MTT solution (500 μg/ml, Amresco, Radnor, PA, U.S.A.) was added and incubated in darkness at 37°C for 8 h. The cells were then treated with DMSO (50 μl) and the absorbance was determined at 550 nm using a SpectraMax M5 Molecular Devices (San Jose, California, U.S.A.).

### Cell cycle analysis

The OECM-1 cell line was seeded in a 10 cm dish at a density of 1 × 10^6^ cells and treated with JCo (40, 60 and 80 μg/ml for 6, 12, 24 and 48 h, respectively). After JCo treatment, cells were collected by centrifugation and stained with propidium iodide (PI) mixed with RNase reagents (Sigma, Missouri, MO, U.S.A.) overnight. Flow cytometric analysis of the stained cells was performed with a Becton-Dickinson FACScan instrument (Franklin Lakes, NJ, U.S.A.), and the data analysis was performed with FlowJo 7.6.1 software (Ashland, Oregon, U.S.A.).

### TUNEL assay

OECM-1 cells were treated with JCo (30 μg/ml) for 24 h and smeared on slides upon collection. After fixation, cells were stained with the In Situ Cell Death Detection Kit, POD (Roche, Mannheim, Germany) according to the manufacturer’s instructions. The slides were visualized immediately under a fluorescence microscope (ZEISS AXioskop2, Carl Zeiss, Thornwood, N.Y., U.S.A.) at 400× magnification to detect apoptotic cells.

### Western blotting analysis

After exposure to JCo, the OECM-1 cells were collected and the total protein was extracted. The samples were subjected to 12% sodium dodecyl sulfate polyacrylamide gel electrophoresis, transferred onto polyvinylidene difluoride membranes, and incubated with primary antibodies overnight. The samples were then incubated at room temperature with anti-rabbit or anti-mouse IgG secondary antibodies for 2 h, and horseradish peroxidase for 1 h. The proteins were detected with an enhanced chemiluminescence detection kit (T-Pro Biotechnology, New Taipei County, Taiwan) and signals were captured using the ImageQuant LAS 4000 image reader (GE LAS-4000, Little Chalfont, U.K.). Primary antibodies involved of p-Rb, cdk2, cdk4, cyclinA, FAS, FASL, bax, bcl-2, caspase-8, and caspase-9 were purchased from Santa Cruz Biotechnology, Inc. (CA, U.S.A.) with usage of 1:200 dilution. The cyclinB1, cyclinD1 and caspase-3 with recommend dilution was purchased from iReal Biotechnology Co., Ltd. (Hsinchu, Taiwan).

### Evaluation of the JCo and 5-FU combination regimes

MTT growth assays were used to evaluate JCo in combination with 5-FU. The cells were seeded in a 96-well overnight and treated with JCo (0–80 μg/ml), 5-FU (0–2 μg/ml), respectively, and JCo plus 5-FU for 24 and 48 h, respectively. The results from the combination assays were analyzed using the combination index method [27,28]. Combination indices (CI) <0.9 are indicative of a synergistic effect between the two agents.

### Statistical analyses

The data are presented as mean ± SD and were applied to determine the significance of differences among different groups with Student’s *t*-test. The differences were considered statistically significant when *P*<0.05.

## Results

### Effects of JCo on the proliferation of OECM-1 cells

**Figure 1:**
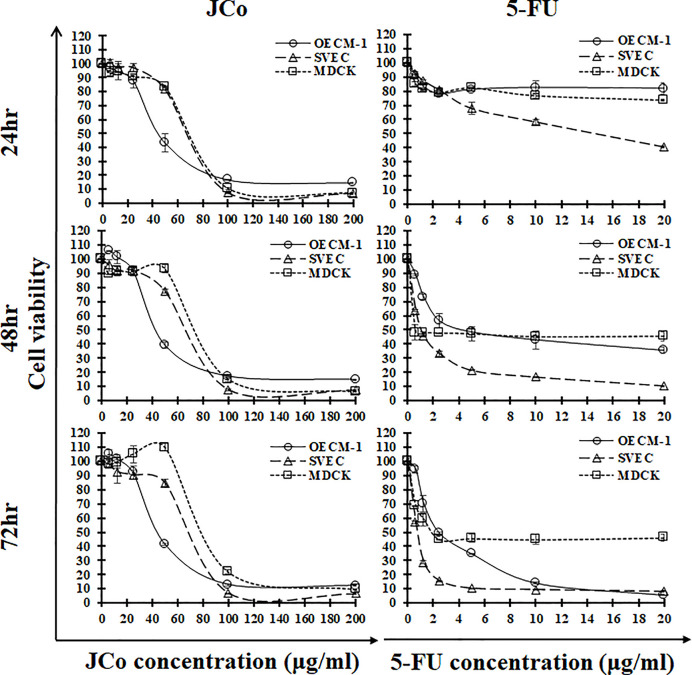
Anti-proliferative activity of JCo on the three cell lines OECM-1, SVEC and MDCK cells were treated with JCo (0–200 μg/ml) or 5-FU (0–20 μg/ml) for 24, 48 and 72 h, and the cell viability was determined using the MTT assay. The data are shown as mean ± SD.

To further test the proliferative inhibitory effect of JCo on human oral squamous cancer cells, the MTT assay was performed. OECM-1 cells were exposed to serial concentrations of JCo for the allocated time periods. As Figure 1 shows, JCo presented a long-term inhibitory effect on OECM-1 cell growth within 72 h in a dose-dependent manner. After JCo treatment, normal cells were inhibited, however, the inhibitory effects of JCo were mitigated with increasing time. 5-FU treatment had a marked inhibitory effect on both tumor and normal cells. The IC_50_ of the OECM-1, SVEC and MDCK cells exposed to JCo for 24 h was 46.20 ± 2.71, 71.45 ± 0.25 and 73.00 ± 0.24 μg/ml, respectively (Table 1). These results indicated that the inhibitory effects of JCo had less impact on normal cells than on cancer cells. The current chemodrug, 5-FU, exhibited a notable IC_50_ value for the OECM-1 and normal cells, and the strong cytotoxic activity of 5-FU was observed not only in the tumor cells, but also in the normal cells. Thus, JCo had an anti-proliferative effect on the OECM-1 cells and might be less cytotoxic to normal cells than 5-FU.

**Table 1:** The IC_50_ of JCo and 5-FU in OECM-1 and normal cells

Cell line	Tumor type		JCo	5-FU
**Oral cancer cell line**				
OECM-1	Human oral squamous cancer cell	24 h	46.20 ± 2.71^a,b^	>20
		48 h	44.87 ± 1.23^a,b,c^	4.52 ± 0.51
		72 h	45.83 ± 0.03^a,b,c^	2.44 ± 0.07
**Normal cell line**				
SVEC	Mouse endothelial cell	24 h	71.45 ± 0.25	14.66 ± 0.57
		48 h	69.64 ± 0.67	1.10 ± 0.01
		72 h	72.45 ± 0.97	0.78 ± 0.03
MDCK	*Canis* epithelial cell	24 h	73.00 ± 0.24	>20
		48 h	77.58 ± 0.45	<0.625
		72 h	84.01 ± 0.33	2.04 ± 0.13

Note: Values were mean ± SD (μg/ml) at 24, 48 and 72 h. a = significant difference between OECM-1 and SVEC in the JCo treatment group (*P*<0.05); b = significant difference between OECM-1 and MDCK in the JCo treatment group (*P*<0.05); c = significant difference between JCo and 5-FU in the OECM-1 group (*P*<0.05).

### Effects of JCo on cell cycle distribution of OECM-1 cells

**Figure 2:**
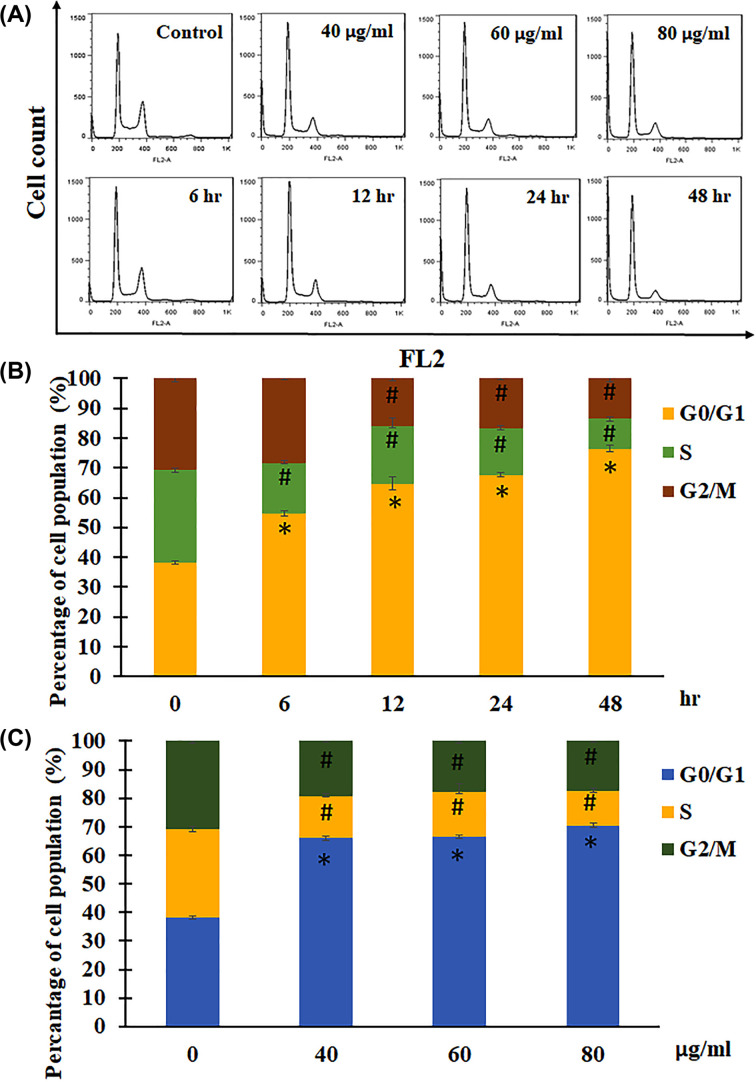
Flow cytometric analysis of cell cycle in OECM-1 cells OECM-1 cells were treated with JCo for the allocated time period, stained with PI, and analyzed using flow cytometry. (**A**) Cell cycle distribution, (**B** and **C**) cell cycle statistical data. *, #: It was significant difference increasing or decreasing between treating and non-treating groups (*P*<0.05).

In order to examine the inhibitory effects of JCo, cell cycle distribution was analyzed after JCo exposure. OECM-1 cells were treated with various concentrations of JCo for the allocated time periods and the cell cycle distribution was analyzed using flow cytometry (Figure 2A). As shown in Figure 2B, the cell population of the G_0_/G_1_ phase was 38.23 ± 0.66%, 54.70 ± 0.76%, 64.65 ± 2.23%, 67.67 ± 0.67% and 76.37 ± 1.06% at the respective time points. As the cell population of the G_0_/G_1_ phase increased, the S and G_2_/M phases decreased. Moreover, after JCo treatment (0, 40, 60 and 80 μg/ml) for 24 h, the data showed a similar trend in the G_0_/G_1_ phase ratio (38.23 ± 0.66%, 66.04 ± 0.84%, 66.67 ± 0.57% and 70.52 ± 0.60%), and both the S phase and G_2_/M phase were diminished after JCo treatment (Figure 2C). Thus, JCo inhibited cell proliferation through induction of cell cycle arrest at the G_0_/G_1_ phase with a reduction in the S and G_2_/M phases.

### Effect of JCo on the expression of cell cycle regulatory proteins

**Figure 3:**
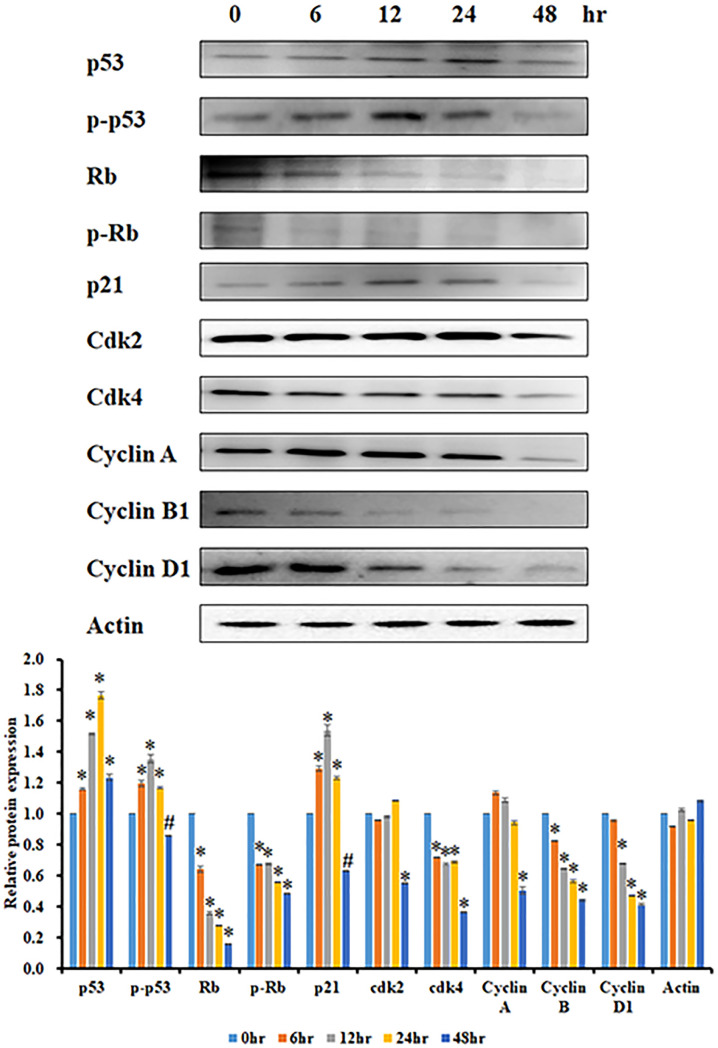
Effects of JCo on the expression of cell cycle-related proteins The cells were treated with JCo (60 μg/ml) for the allocated time period and then lysed by western blotting to detect protein expression. *, #: It was significant difference increasing or decreasing between treating and non-treating groups (*P*<0.05).

The inhibitory effects of JCo on the expression of proteins involved in cell cycle regulation were examined. The OECM-1 cells were treated with JCo (60 μg/ml) for 0, 6, 12, 24 and 48 h, and the expression levels of relevant proteins were analyzed by Western blotting. The p53 and retinoblastoma protein (Rb) are tumor suppressors that can mediate cell cycle progression. Rb is also a key regulatory protein in cell cycle progression, depending on its phosphorylation state. The p53 and phosphorylated p53 protein levels increased after JCo treatment, as did increasing of the p21 level, which is a protein of p53 downstream. CyclinA/B1/D1 and Cdk2/4 proteins, which participate in cell cycle progression, declined with time, as shown in Figure 3. In addition, Rb and phosphorylated Rb decreased in a time-dependent manner. Collectively, these results indicate that JCo recued OECM-1 cell proliferation through the regulation of key cell cycle proteins, thereby arresting the cell cycle at the G_0_/G_1_ phase.

### JCo-induced OECM-1 cell apoptosis

**Figure 4:**
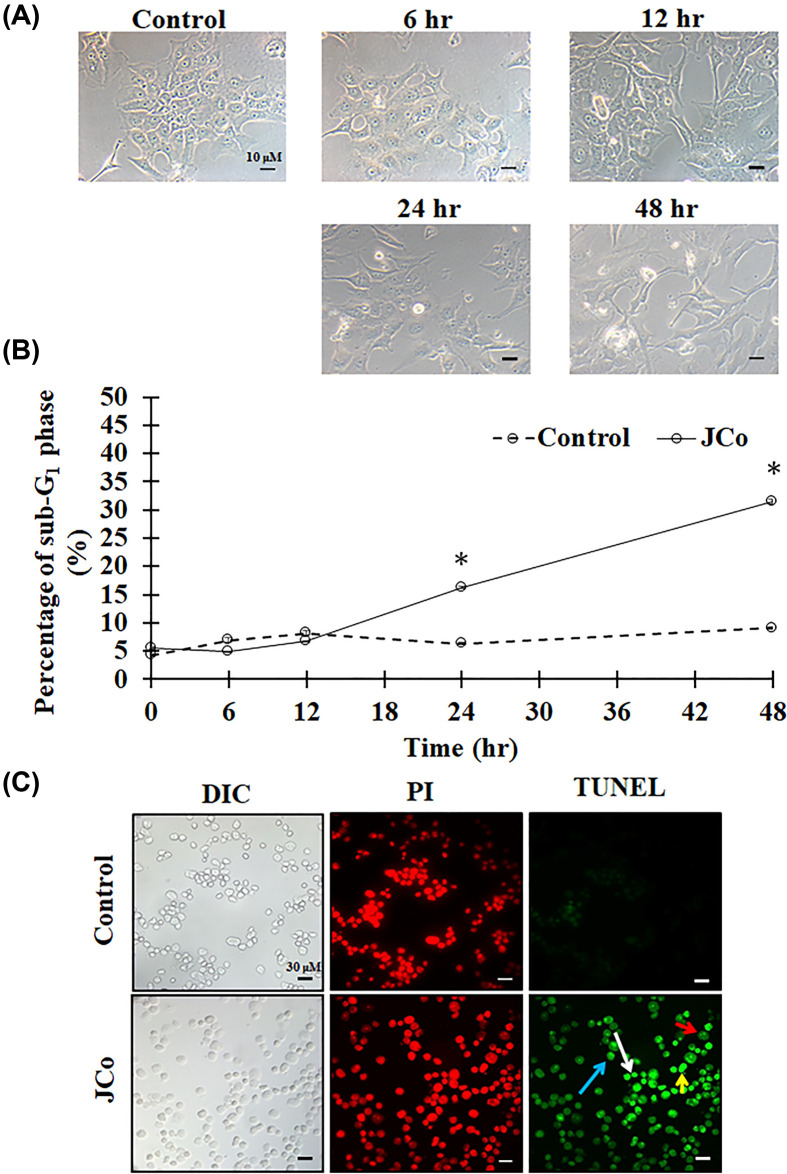
Apoptotic effects of JCo in OECM-1 cells After JCo exposure, cells were collected and observed. (**A**) The morphology of OECM-1 cells. (**B**) The sub-G_1_ phase of the cell population. *: It was significant difference increasing between control and treating groups (*P*<0.05). (**C**) The cells were stained with PI (red) and TUNEL (green) reagents; white arrows indicate apoptotic bodies; red arrows indicate anoikis; blue arrows indicate chromatin condensation; yellow arrows indicate DNA fragments.

After JCo exposure, numerous effects on the morphology of the OECM-1 cells were observed, such as shrinkage, elongation, detachment and death (Figure 4A). In addition, JCo treatment increased the cell population in the sub-G_1_ phase in a time-dependent manner, suggesting that JCo-induced cell death (Figure 4B). The TUNEL assay was conducted to test whether JCo causes cell apoptosis. The results showed that JCo-induced cell apoptotic properties, including apoptotic bodies, chromatin condensation, anoikis and DNA fragments (Figure 4C). Thus, JCo stimulated cytotoxic activity in OECM-1 cells through induction of cell apoptosis.

### Effect of JCo on the expression of apoptotic proteins

**Figure 5:**
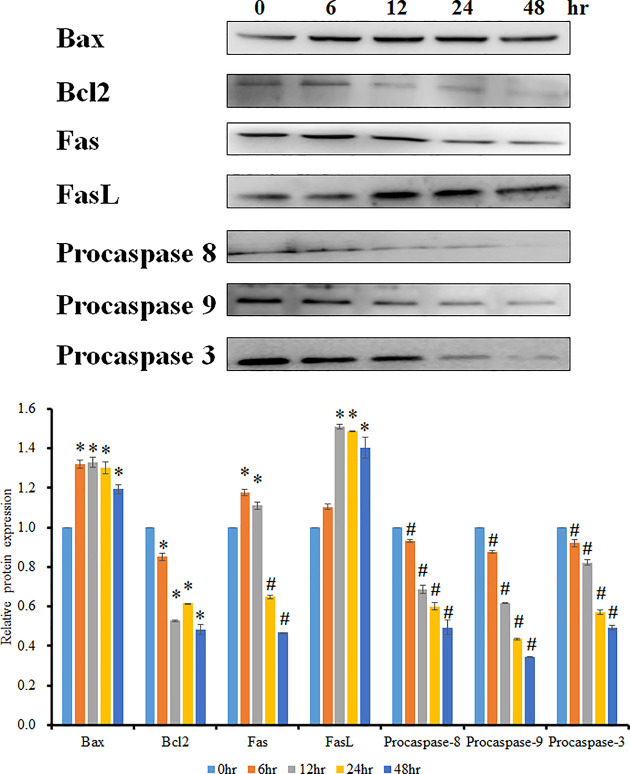
Effects of JCo on the expression of cell apoptosis pathway proteins The OECM-1 cell line was treated with JCo (60 μg/ml) for the allocated time periods and then lysed by Western blotting to detect protein expression. *, #: It was significant difference increasing or decreasing between treating and non-treating groups (*P*<0.05).

In order to explore the cell apoptosis pathway after JCo treatment, cell apoptosis-related proteins were examined. As shown in Figure 5, the protein level of bax increased, whereas bcl2 decreased; in other words, the bax/bcl2 ratio increased, suggesting that mitochondrial membrane potential imbalance for the release of cytochrome *c* results in caspase-9 cleavage and activation of the intrinsic apoptotic pathway. Moreover, the FAS protein level decreased, FASL increased, and downstream pro-caspase-8 declined, revealing that the extrinsic apoptotic pathway was activated. The key mediator protein, caspase-3, decreased in a time-dependent manner. These results reveal that JCo induced not only the intrinsic apoptotic pathway, but also the extrinsic apoptotic pathway leading to OECM-1 cell death.

### JCo combination of 5-FU

**Figure 6:**
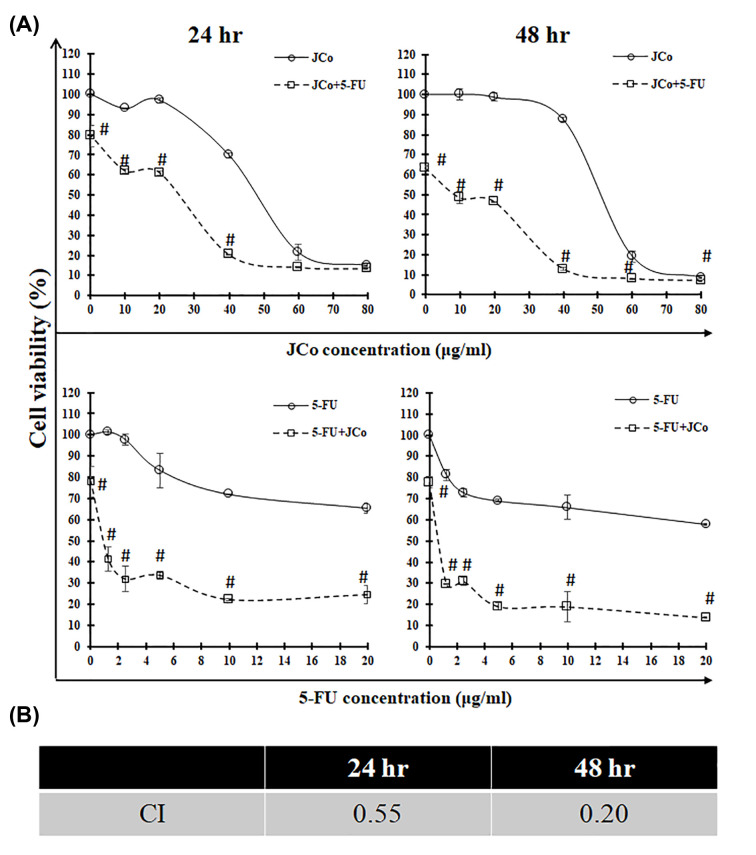
JCo combined with 5-FU The cells were plated and subjected to a combination of JCo and 5-FU. After 24 and 48 h incubation, removing the drugs, adding MTT solution and dissolving with DMSO, the cell viability was measured using the MTT assay. (**A**) The cell viability of JCo combined with 5-FU. (**B**) The CI values of JCo combined with 5-FU in 24 and 48 h. Results are shown as mean ± SD. Data were compared for each drug separately and taken together using the Student’s *t*-test. Significant differences (#*P* < 0.05) were observed in the two drugs concerning cytotoxicity.

To study the synergistic effect between JCo and 5-FU, the MTT assay was performed to evaluate the combination index. OECM-1 cells were treated with JCo and 5-FU at the same time for 24 and 48 h. As shown in Figure 6, JCo combined with 5-FU markedly reduced cell growth compared with treatment of JCo or 5-FU alone. Furthermore, the combination index of JCo combined with 5-FU at 24 and 48 h was 0.55 and 0.20, respectively, suggesting that JCo combined with 5-FU exhibited a synergistic effect to inhibit OECM-1 cells.

## Discussion

*Juniperus communis* has been shown to activate apoptotic cell death in different tumorigenic cell lines. In the present study, we found that JCo was effective at suppressed oral cancer cell growth without affecting normal cells in the same way. The normal cells used in the present study were endothelial and kidney cells, and the IC_50_ of these cells was higher than in the OECM-1 cells after JCo treatment, ranging from 1.5- to 1.8-fold, which suggested that JCo efficiently inhibited oral cancer cell growth but was less toxic to normal cells. Moreover, the results revealed that 5-FU had higher cytotoxicity than JCo in normal cells, suggesting that JCo might have fewer side effects *in vivo* when conducted experiments with animals.

When analyzing cell cycle distribution to understand how JCo affects cell proliferation, we found that JCo induced cell cycle arrest at the G_0_/G_1_ phase, with a reduction in the S and G_2_/M phases. Therefore, we investigated the mechanisms of JCo-regulated cell cycle progression. The cell cycle is regulated by tumor suppressors, such as p53 and Rb; however, tumor suppressor genes are commonly mutated in cancer cells and cause uncontrolled cell proliferation. p53 is one of the tumor suppressors that is activated to block the cell cycle process when DNA is damaged. Our data showed that p53 was phosphorylated to activate the downstream molecule, p21, which is a cyclin-dependent kinase inhibitor that can block the cell cycle at any phase after JCo treatment [29,30]. Another tumor suppressor gene, Rb, controls cell cycle progression by phosphorylation [31], and the results showed that the levels of Rb and p-Rb were diminished after JCo treatment. Taken together, these results demonstrate that JCo might trigger phosphorylation of p53, de-phosphorylation of Rb, and activation of p21, consequently affecting the expression of relevant cell cycle proteins that contribute to cell cycle arrest at the G_0_/G_1_ phase.

Apoptosis is a common area of study for cancer therapy. In the present study, we found that JCo caused oral cancer cell death. In order to detect whether JCo induces OECM-1 cell apoptosis, we first observed the change in cell morphology before checking the cell population in the sub-G_1_ phase, and finally the classic cell death morphology by TUNEL assay. All these investigations demonstrated that JCo does indeed contribute to the induction of OECM-1 cell apoptosis. Hence, we explored the apoptotic pathway induced by JCo. The apoptotic pathway can be divided in two; one is the intrinsic apoptotic pathway, often induced by mitochondrial membrane potential loss and affecting bax and bcl2 protein expression, resulting in procaspase-9 cleavage; the other is the extrinsic apoptotic pathway, which is activated by death ligand binding to death receptors, such as FAS and FASL, and triggers the cleavage of procaspase-8. Caspase-3 is then cleaved, which stimulates caspase cascade activation and, subsequently, cell apoptosis. The data revealed that JCo effectively induced the intrinsic and extrinsic apoptotic pathways, contributing to OECM-1 cell apoptosis.

Combination therapy is a common practice in oral cancer therapy [32,33]; however, it is associated with many side effects in patients. Therefore, we explored whether JCo combined with 5-FU exhibited a synergistic effect to reduce cytotoxicity in normal cells and enhance the inhibitory effect on oral cancer cells. Our data demonstrated that JCo had a significant synergistic effect with 5-FU, with a combination index <1. Moreover, the results indicate that less usage of 5-FU and JCo results in a better proliferative inhibitory function in oral cancer cells.

## Conclusions

JCo inhibited oral cancer cell growth by inducing cell cycle arrest and cell apoptosis. Additionally, JCo combined with 5-FU had synergistic effects in treating oral cancer. As a result, JCo might provide a new therapeutic strategy and represented a potential candidate for chemotherapy of oral cancer.

## Abbreviations

5-FU: 5-fluorouracil
JCo: *Juniperus communis*
PI: propidium iodide
